# Ablation of Gadd45β ameliorates the inflammation and renal fibrosis caused by unilateral ureteral obstruction

**DOI:** 10.1111/jcmm.15519

**Published:** 2020-06-22

**Authors:** Sung‐Je Moon, Jae‐Hoon Kim, Young‐Keun Choi, Chul‐Ho Lee, Jung Hwan Hwang

**Affiliations:** ^1^ Laboratory Animal Resource Center Korea Research Institute of Bioscience and Biotechnology (KRIBB) Daejeon Korea; ^2^ University of Science and Technology Daejeon Korea

**Keywords:** chronic kidney disease, inflammation, renal fibrosis, TGF‐β signalling, unilateral ureteral obstruction

## Abstract

The growth arrest and DNA damage‐inducible beta (Gadd45β) protein have been associated with various cellular functions, but its role in progressive renal disease is currently unknown. Here, we examined the effect of Gadd45β deletion on cell proliferation and apoptosis, inflammation, and renal fibrosis in an early chronic kidney disease (CKD) mouse model following unilateral ureteral obstruction (UUO). Wild‐type (WT) and Gadd45β‐knockout (KO) mice underwent either a sham operation or UUO and the kidneys were sampled eight days later. A histological assay revealed that ablation of Gadd45β ameliorated UUO‐induced renal injury. Cell proliferation was higher in Gadd45β KO mouse kidneys, but apoptosis was similar in both genotypes after UUO. Expression of pro‐inflammatory cytokines after UUO was down‐regulated in the kidneys from Gadd45β KO mice, whereas UUO‐mediated immune cell infiltration remained unchanged. The expression of pro‐inflammatory cytokines in response to LPS stimulation decreased in bone marrow‐derived macrophages from Gadd45β KO mice compared with that in WT mice. Importantly, UUO‐induced renal fibrosis was ameliorated in Gadd45β KO mice unlike in WT mice. Gadd45β was involved in TGF‐β signalling pathway regulation in kidney fibroblasts. Our findings demonstrate that Gadd45β plays a crucial role in renal injury and may be a therapeutic target for the treatment of CKD.

## 1 INTRODUCTION

The prevalence and burden of chronic kidney disease (CKD) are rising worldwide, and obstructive renal injury is the most common cause for renal fibrosis.^1^, ^2^ Renal fibrosis is the key process underlying the progression of CKD to end‐stage renal disease and is the outcome of an excessive amassment of extracellular matrices that occurs in CKD.^2^ Glomerulosclerosis, tubulointerstitial fibrosis and excess accumulation of matrices in the interstitial site surrounding tubules and peritubular capillaries are common findings in patients with renal fibrosis.^3^ The pathogenesis of CKD is primarily prominent renal hemodynamic and metabolic changes, tubular damage and cell death caused by apoptosis or necrosis, leading to increased infiltration of immune cells into the renal interstitium.^4^ Although transforming growth factor‐β (TGF‐β) is considered the master regulator that culminates in fibrosis and renal parenchymal loss, the importance of genetic and epigenetic factors is accepted.

TGF‐β is a prototypic fibrogenic growth molecule that controls various biological actions, including cell growth, differentiation and immunological reactions.^5^, ^6^ In renal fibrosis, one of the most important actions of TGF‐β is the adjustment of ECM accumulation via inhibiting matrix breakdown by declining the synthesis of metalloproteinases and increasing the synthesis of their inhibitors.^7^ In human glomerular diseases, up‐regulated TGF‐β expression occurs in progressive glomerular diseases, and this expression is strongly correlated with fibrotic areas in biopsy specimens.^8^ The expression of TGF‐β receptors is also enhanced in diseased glomeruli.^8^ The binding of TGF‐β to its cognate receptors, such as TGF‐β type I and II receptors, results in the stimulation of canonical and non‐canonical intracellular signalling pathways.^9^, ^10^ Smad2 and Smad3 are the main molecules involved in canonical pathways activated by TGF‐β to form a complex with Smad4, which then translocate into the nucleus and regulate the transcription of the target genes.^11^ Additionally, Smad6 and Smad7 are known as negative regulators of Smad2/3, and thereby inhibit TGF‐β signalling.^12^, ^13^ Recently, it was found that Gadd45β is a novel protein that regulates the TGF‐β signalling pathway by blocking the Smurf‐induced breakdown of TGF‐β receptor type 1 in colon tissue and intestinal epithelial cell lines.^14^ Gadd45β is a small protein included in the Gadd45 family that is composed of Gadd45α, Gadd45β and Gadd45γ.^15^ In addition to the regulation of TGF‐β signalling, we and others have previously shown that Gadd45β regulates DNA repair, cell cycle arrest, cell proliferation, apoptosis and inflammation, depending on the cell type.^16^, ^17^ However, the roles of Gadd45β in CKD remain unclear.

In the current study, we investigated the effects of Gadd45β ablation on cell survival, apoptosis, inflammation and renal fibrosis in mice. The unilateral ureteral obstruction (UUO) model was used to induce renal fibrosis and immune cell infiltration and to estimate the susceptibility against UUO‐induced nephropathy.

## 2 MATERIALS AND METHODS

### 2.1 Antibodies and reagents

Antibodies (Abs) against phosphorylated‐Akt (pS473), total Akt, phosphorylated‐Smad2, total‐Smad2/3, phosphorylated‐Smad3, total Smad7, phosphorylated extracellular‐signal‐regulated kinase (pERK)1/2, total ERK1/2, cleaved caspase‐3 and α‐tubulin were purchased from Cell Signaling. An Ab against Gadd45β was obtained from Aviva Systems Biology. Ab against β‐actin was purchased from Santa Cruz Biotechnology. Abs against CD3, alpha‐smooth muscle actin (α‐SMA), TGF‐β receptor type I (TGF‐βRI) and Ki‐67 were purchased from Abcam. An Ab against F4/80 was purchased from Bio‐Rad Laboratories. Human recombinant TGF‐β1 was purchased from Sigma.

### 2.2 Animals

A whole‐body Gadd45β knockout (KO) mouse under a C57BL/6 background was purchased from Jackson Laboratory. All mice were maintained in a room maintained at constant temperature (20‐22°C) on a 12:12 hours light:dark cycle with free access to food and water under specific pathogen‐free conditions. For the UUO model, Gadd45β wild‐type (WT) and KO male mice were anaesthetized with avertin (200 mg/kg) and placed on a hot pad (34°C) to prevent hypothermia. After anaesthesia, the anterior abdominal skin was shaved and wiped with 70% ethanol. The left ureter was exposed and ligated twice using a nylon suture. Sham‐operated mice without ligation were prepared as controls. Mice were killed by cervical dislocation, and the kidney tissues were removed at eight days after UUO for molecular analysis and histological examination. All animal experiments were approved by the Institutional Animal Care and Use Committee (KRIBB‐AEC‐19254) and were performed in accordance with the institutional guidelines of the Korea Research Institute of Bioscience and Biotechnology.

### 2.3 Cell culture and transfection

Human embryonic kidney HEK293T cells were cultured in Dulbecco's Modified Eagle Medium (DMEM; HyClone) supplemented with 10% foetal bovine serum (FBS, HyClone) and 100 U/mL penicillin and 0.1 mg/mL streptomycin (Gibco). HK‐2 cells, a proximal tubule epithelial cell line from normal adult human kidney, were maintained in DMEM/F‐12 (50/50 mix) (Corning) supplemented with 10% FBS and penicillin and streptomycin (Gibco). For the overexpression of proteins, cells were plated in a six‐well culture dish and transfected with 500 ng/well Gadd45β plasmid DNA14 or the same concentration of an empty vector using Lipofectamine LTX reagent with Plus according to the manufacturer's protocol (Thermo Fisher Scientific). For Gadd45β silencing, cells were cultured in six‐well plates and transfected with siRNA for Gadd45β using Lipofectamine RNAiMAX, according to the manufacturer's instructions (Thermo Fisher Scientific). A scrambled siRNA was used as a negative control (Thermo Fisher Scientific). The sequences of human Gadd45β cDNA were used as follows: 5′‐ACGAGUCGGCCAAGUUGAUGAAUGU‐3′, 5′‐CAGUCCUUCUGCUGUGACAACGACA‐3′ and 5′‐GAGGUGGCCAGCUACUGCGAAGAAA‐3′.

### 2.4 Cell stimulation with TGF‐β1

The cells were transfected with V5‐tagged Gadd45β or empty vector for one day, washed with PBS, and subsequently stimulated with 10 ng/mL TGF‐β1 for the indicated duration. For the silencing of Gadd45β, cells were plated in a six‐well plate (1 × 10^5^) and transfected with siRNA for the control or Gadd45β for three days. Silenced cells were further stimulated with or without 10 ng/mL TGF‐β1 for the indicated duration.

### 2.5 Isolation and differentiation of mouse bone marrow cells

Male WT and Gadd45β KO mice were killed by cervical dislocation, and both femurs were removed. Bone marrow was flushed using PBS; the flushed cells were gently passed through a 70 μm Falcon™ Cell Strainer (Life Sciences) and harvested by centrifugation at 220 *g* for 5 minutes. The harvested cells were washed with PBS, re‐suspended with complete medium, and cultured in a petri dish. Some attached cells were eliminated after overnight culture. The non‐adherent cells were harvested into a new tube and washed with PBS three times. Next, bone marrow cells were stimulated with M‐CSF (R&D system) for differentiation to bone marrow‐derived macrophages (BMDMs) as reported previously.^18^ Briefly, the bone marrow cells were stimulated with 20 ng/mL M‐CSF. After seven days of culturing, BMDMs were obtained with >90% purity.

### 2.6 LPS stimulation of BMDMs and HK‐2 cells

Isolated BMDMs (1 × 10^6^ cells/well) were seeded into a six‐well plate and incubated overnight. These BMDMs were further incubated with 100 ng/mL LPS or PBS for the indicated time. HK‐2 cells were treated with scrambled siRNA or Gadd45β siRNA for two days and further stimulated with 1 μg/mL LPS or the same volume of PBS for the indicated time. Then, stimulated cells were washed with PBS twice, collected and lysed for further analysis.

### 2.7 Renal histological analysis

The kidney tissues fixed in 10% neutral buffered formalin were embedded in paraffin and cut to 4 μm‐thick sections. The sections were deparaffinized and stained with haematoxylin & eosin (H&E), Sirius red and Masson trichrome according to the standard protocol. Blinded analysis of kidney tissues was performed using a light microscope. A board‐certified pathologist examined the kidney tissue tubular injury scores as follows: 0 = normal kidney; 1 = minimal (≤10% involvement); 2 = mild (10%‐25% involvement); 3 = moderate (26%‐50% involvement); 4 = severe (51%‐75% involvement); and 5 = very severe (>75% involvement).

### 2.8 Immunohistochemistry

Kidney slides (with 4‐μm thick sections) were deparaffinized in sequential steps of xylene, ethanol, and finally rehydrated in water. Antigen retrieval was performed by immersing the samples in boiling sodium citrate buffer (20 mmol/L sodium citrate, 0.05% Tween 20, pH 6.5) in a microwave for 15 minutes. The tissues were blocked with normal serum and incubated with primary antibodies against Ki‐67, cleaved caspase 3, CD3 and F4/80 overnight at 4°C. Next, the slides were washed with PBS three times and further incubated with secondary antibodies (Vectastain Elite HRP ABC kit, Vector Laboratories) for 1 hour at 37°C. Positive signals were imaged by 3,3‐diaminobenzidine (DAB, Peroxidase substrate kit, Vector Laboratories). Eventually, the tissue samples were counterstained with haematoxylin. ImageJ software was used to calculate the area of interest.

### 2.9 RNA isolation and quantitative real‐time polymerase chain reaction (qRT‐PCR)

Total RNA from kidney tissues were isolated using TRIzol reagent according to the manufacturer's instructions (Thermo Fisher Scientific). RNA concentrations were determined spectrophotometrically. Double‐stranded cDNA was synthesized using the iScript™ cDNA Synthesis Kit (Bio‐Rad). The resulting cDNA was subjected to qRT‐PCR using the StepOnePlus™ Real‐Time PCR system (Applied Biosystems) with AccuPower^®^ 2× Greenstar qPCR Master Mix according to the standard protocol (Bioneer). A list of primer sequences is provided in Table 1. The expression of the target genes was normalized by 18S rRNA.

**Table 1:** List of primers of each gene used for quantification by qRT‐PCR

Gene name	Direction	Primer sequence
mCD3	Forward	TGCCCTCTAGACAGTGACGA
	Reverse	TTGAGGCTGGTGTGTAGCAG
mF4/80	Forward	GATGAATTCCCGTGTTGTTG
	Reverse	ACATCAGTGTTCCAGGAGAC
mIL‐1β	Forward	AGGAGCTATCACTTGACCACAT
	Reverse	TGATGTGCTGCTGCGAGATT
hIL‐1β	Forward	GGACAAGCTGAGGAAGATGC
	Reverse	TCGTTATCCCATGTGTCGAA
mIL‐6	Forward	TCCATCCAGTTGCCTTCTTG
	Reverse	TTCCACGATTTCCCAGAGAAC
mTNFα	Forward	CCCTCACACTCAGATCATCTTCT
	Reverse	GCTACGACGTGGGCTACAG
miNOS	Forward	GTTCTCAGCCCAACAATACAAGA
	Reverse	GTGGACGGGTCGATGTCAC
mIL‐4	Forward	AACGAGGTCACAGGAGAAGG
	Reverse	TCTGCAGCTCCATGAGAACA
mIL‐5	Forward	GAAGTGTGGCGAGGAGAGAC
	Reverse	GCACAGTTTTGTGGGGTTTT
mIL‐10	Forward	GGGTTGCCAAGCCTTATCG
	Reverse	TCTCACCCAGGGAATTCAAATG
mIL‐13	Forward	GCAACATCAACAGGACCAGA
	Reverse	GTCAGGGAATCCAGGGCTAC
mCD25	Forward	CCGAGAGTGAGACTTCCTGC
	Reverse	GCTGGCCACTGCTACCTTAT
mCD28	Forward	CGGGAATGGGAATTTTACCT
	Reverse	TTGACGTGCAGATTCCAGAG
mCD38	Forward	CTGGAGAGCCTACCACGAAG
	Reverse	GCAAGGGTTCTTGGAAACAA
mCD69	Forward	ACATCTGGAGAGAGGGCAGA
	Reverse	AAGGACGTGATGAGGACCAC
18s	Forward	GACACGGACAGGATTGACAGATTGATAG
	Reverse	GTTAGCATGCCAGAGTCTCGTTCGTT

### 2.10 Measurement of plasma pro‐inflammatory cytokines

Plasma samples from UUO‐mice were harvested at 8 days after UUO and pro‐inflammatory cytokines such as interleukin 1 beta (IL‐1β), interleukin 6 (IL‐6) and tumour necrosis factor alpha (TNFα) were measured using ELISA kit according to the manufacturer's instructions (BD Biosciences), respectively.

### 2.11 Western blotting

Kidney tissues or cells were lysed in RIPA buffer containing protease inhibitor cocktail (Roche Applied Science). Protein concentrations were quantified by the Bradford's assay method. The equivalent proteins were separated by electrophoresis on a polyacrylamide gel and transferred to a polyvinylidene fluoride membrane. The membranes were incubated with corresponding primary and secondary antibodies. Housekeeping proteins were used as a loading control.

### 2.12 Statistical analysis

Statistical analyses were performed using GraphPad Prism software. All data are presented as mean ± SEM. Stained areas were analysed using ImageJ software, and immunoblot analysis results were quantified using Tina 2.0 software and ImageJ. Two‐group comparisons were performed using two‐tailed Student's *t* test. A *P*‐value of <.05 was considered statistically significant.

## 3 RESULTS

### 3.1 The effect of Gadd45β deficiency in UUO‐induced renal damage

**Figure 1:**
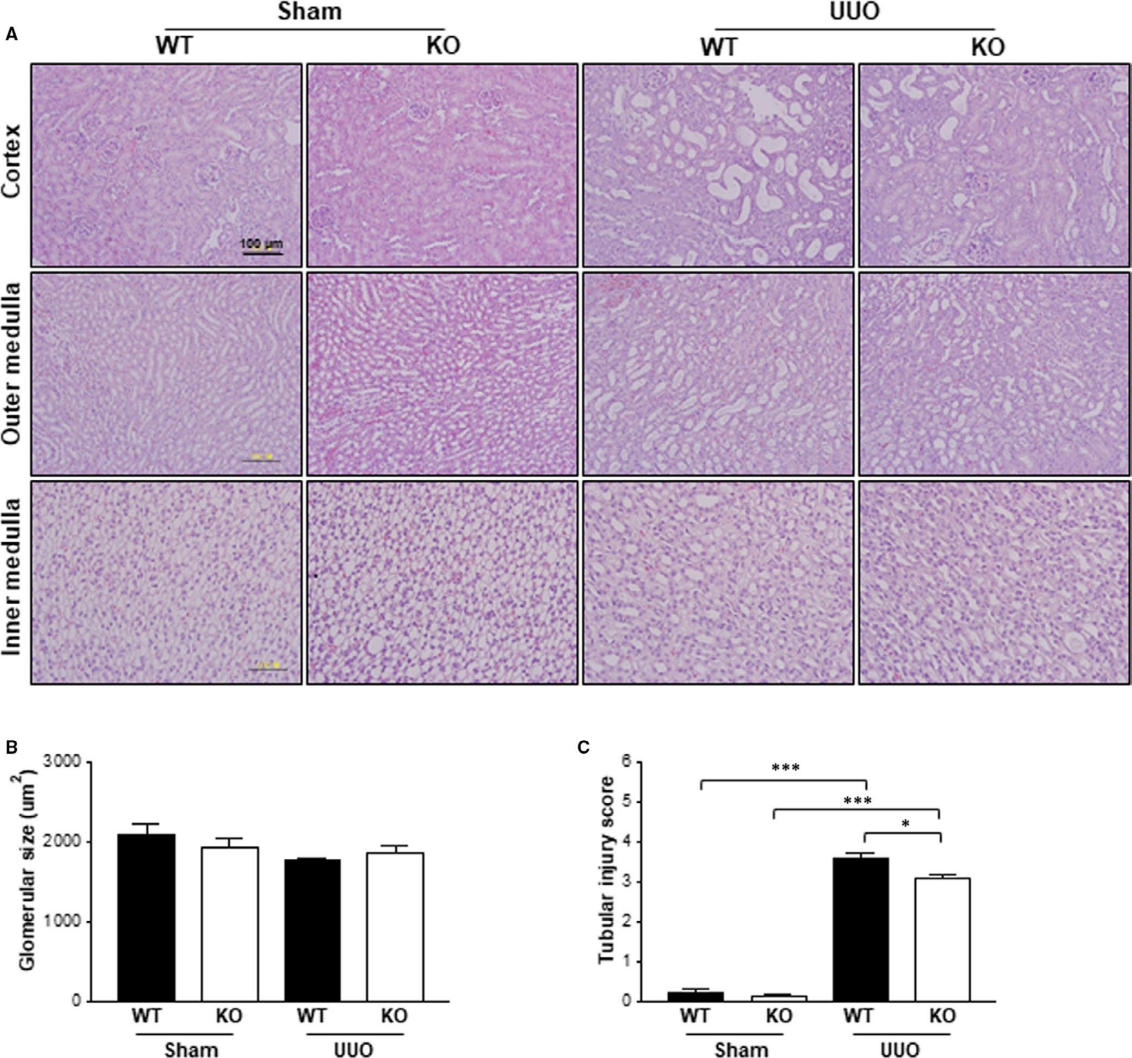
Tubular damage after UUO was increased in Gadd45β KO mouse kidneys compared with those of WT mice. A, Representative kidney sections were stained with H&E to observe glomerular size and tubular injury. All images were taken at a magnification of 20x. The scale bar is 100 μm. B, The glomerular size and (C) tubular injury score were taken from the cortex regions of the H&E stained kidney sections of WT‐sham (n = 4), KO‐sham (n = 4), WT‐UUO (n = 5) or KO‐UUO (n = 6) mice. All values are means ± SEM. *P < .05 or ***P < .001

To investigate the involvement of Gadd45β in acute renal injury, the standard UUO experimental model of renal failure was used and the functional contribution of Gadd45β to the development of tubular injury was assessed by estimating glomerular size and tubular injury score in the sections stained with H&E (Figure 1A). No significant differences in glomerular size between all groups were observed (Figure 1B). However, tubular injury significantly increased after UUO, as reflected by the presence of dilated tubules, loss of brush borders, and epithelial simplification in the tubulointerstitium in Gadd45β WT and KO mice (Figure 1C). Interestingly, Gadd45β‐KO kidneys showed reduced tubular injury compared with those of Gadd45β WT mice (Figure 1C), suggesting that Gadd45β is involved in the development of renal injury.

### 3.2 The effects of Gadd45β deficiency on UUO‐induced cell proliferation and apoptosis

**Figure 2:**
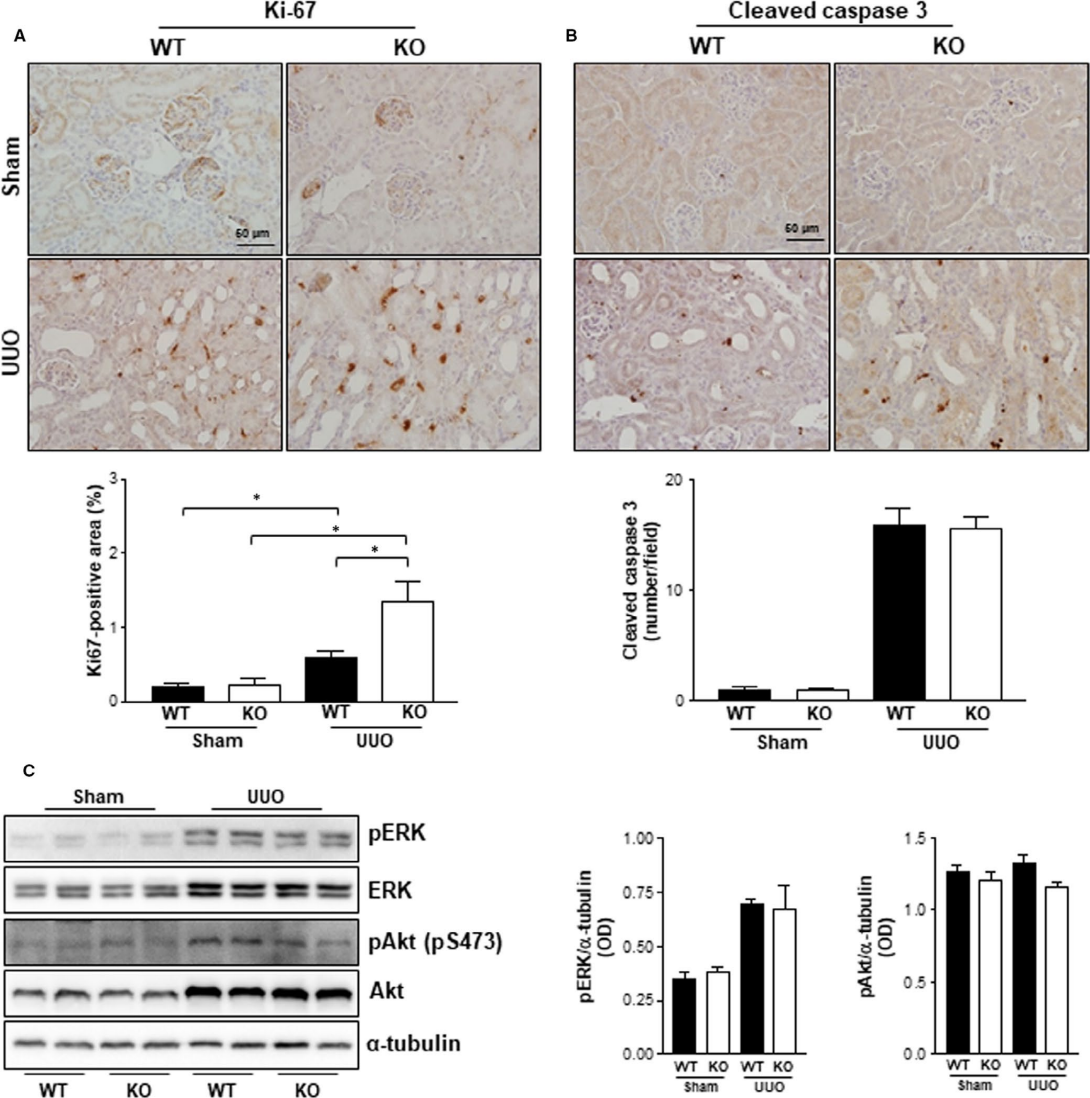
Cell marker staining of cell proliferation and apoptosis. A, Proliferating cells were stained with Ki‐67 antibody. B, Apoptotic cells were stained with cleaved caspase 3 antibody. All images were taken at a magnification of 40×. The scale bar is 50 μm. Positive areas stained for Ki‐67 or cleaved caspase 3 on kidney sections of WT‐sham (n = 4), KO‐sham (n = 4), WT‐UUO (n = 5) or KO‐UUO (n = 6) mice were obtained using ImageJ software. *P < .05. C, Western blotting was performed with specific antibodies of the indicated proteins and optical density was calculated

Renal injury in the UUO model can lead to an imbalance between tubular epithelial cell apoptosis and proliferation. Therefore, immunohistochemistry of kidney tissues was performed using specific antibodies against Ki‐67 or cleaved caspase 3 to determine cell proliferation and apoptosis, respectively. Interestingly, cell proliferation increased by approximately threefold after UUO in Gadd45β WT kidneys and was more prominent in Gadd45β KO kidneys compared with those of the WT mice (approximately sixfold increase) (Figure 2A). Although apoptosis was markedly up‐regulated after UUO, significant differences were not observed in either genotypes (Figure 2B). ERK and Akt are major mediators that regulate cell proliferation. Therefore, the activation of these proteins was estimated by using specific antibodies against active phosphorylated residues on these kinases. The total and phosphorylated levels of these kinases increased after UUO compared with the sham operation, but no significant differences were observed between either genotype, suggesting that another pathway might be involved in cell proliferation, mediated by Gadd45β deficiency (Figure 2C).

### 3.3 The effects of Gadd45β deficiency against UUO‐induced inflammatory cell infiltration

**Figure 3:**
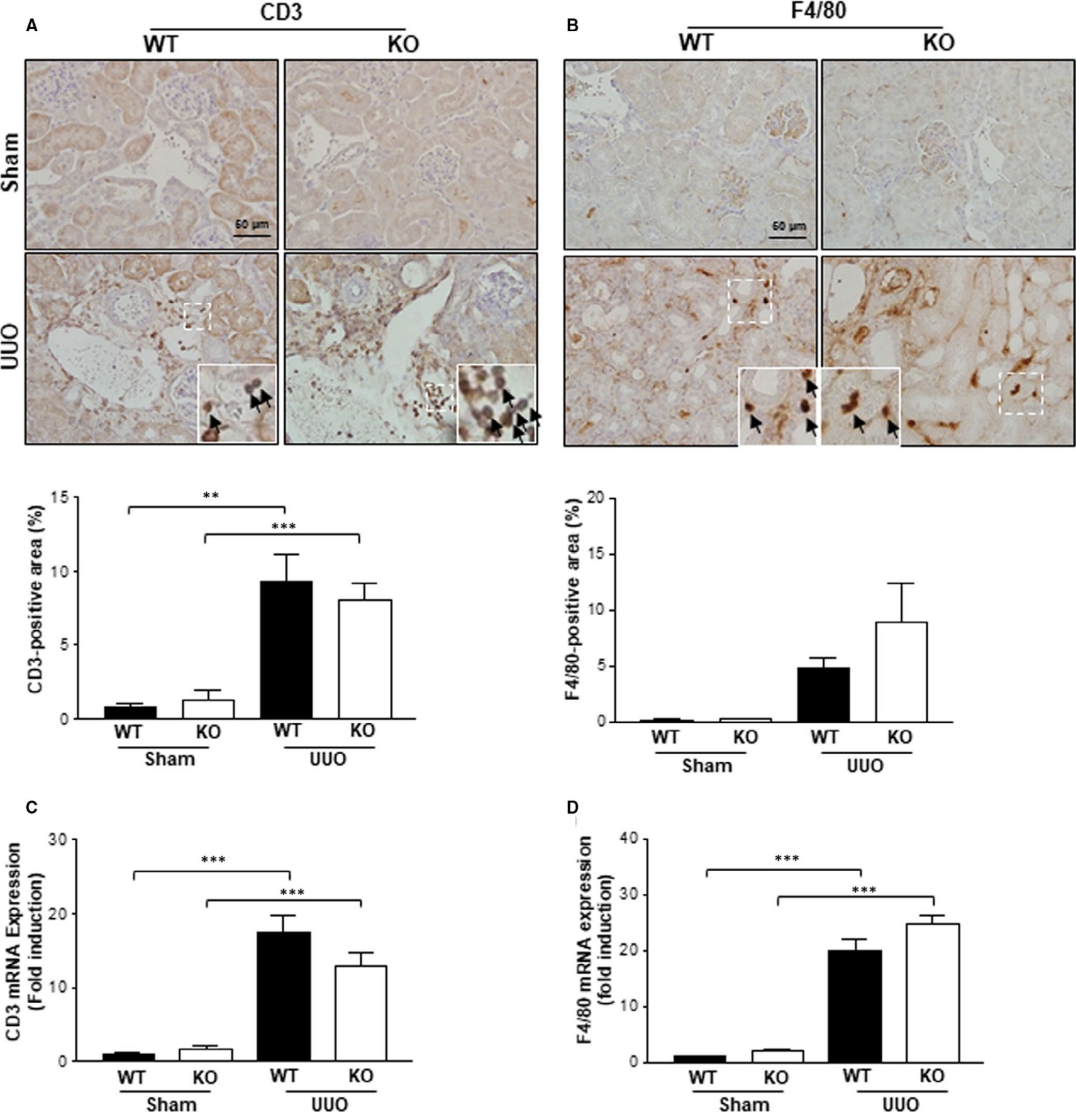
Markers of inflammatory cells in WT and Gadd45β KO kidneys. A, T cells were stained with anti‐CD3 and the positive area was quantified. B, Macrophages were stained with anti‐F4/80 and the positive area was quantified. Renal mRNA levels of CD3 (C) and F4/80 (D) in the sham‐ and UUO‐operated groups. All graphical data were taken from kidney sections of the WT‐sham (n = 4), KO‐sham (n = 4), WT‐UUO (n = 5) or KO‐UUO (n = 6) mice. **P < .01 or ***P < .001

The recruitment of inflammatory cells, such as T‐lymphocytes and macrophages, is an early event in progressive renal injury.^19^ Therefore, whether Gadd45β deficiency could affect renal immune infiltration in the kidneys after UUO was determined. Antibodies against the T‐cell marker CD3 and macrophage marker F4/80 were applied in IHC analyses, respectively. In line with previous studies, UUO caused immune cell infiltration in the kidneys of Gadd45β WT and KO mice, as estimated by the positive area in the respective kidney samples (Figure 3A,B). However, CD3‐and F4/80‐positive areas were similar in the kidneys of Gadd45β KO and WT mice, despite reduced renal injury (Figure 3A,B). The mRNA expression of both markers was further estimated to confirm IHC data. Consistent with the IHC study, no significant differences were observed in either genotypes (Figure 3C,D).

### 3.4 The effects of Gadd45β deficiency against UUO‐induced inflammatory cytokine expression

**Figure 4:**
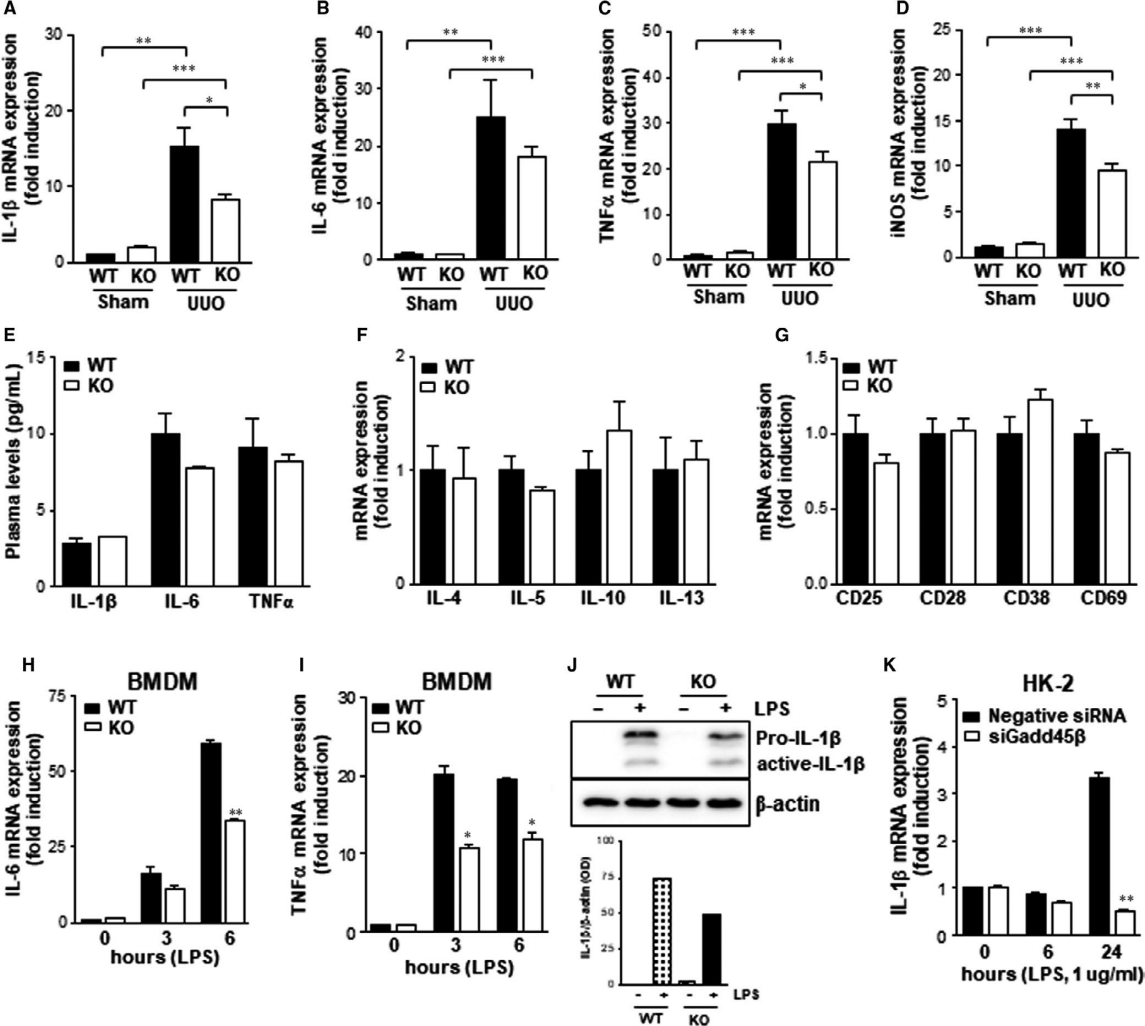
The expression of inflammatory cytokines in vivo and in vitro. Renal mRNA levels of pro‐inflammatory cytokines such as IL‐1β (A), IL‐6 (B), TNFα (C) and iNOS (D) in WT‐sham (n = 4), KO‐sham (n = 4), WT‐UUO (n = 5) or KO‐UUO (n = 6) groups. E, Plasma levels of pro‐inflammatory cytokines were measured by ELISA assay. F, Renal mRNA expression of anti‐inflammatory cytokines. G, The expression of genes related to T‐cell activation. H and I, The mRNA expression levels of IL‐6 and TNFα in response to LPS (100 ng/mL) for the indicated times were determined in BMDMs isolated from WT and Gadd45β KO mice. Data are representative of at least two independent experiments. *P < .05, **P < .01 or ***P < .001. J, Pro‐IL‐1β protein levels were estimated by Western blot analysis in LPS‐treated BMDMs. The protein levels were quantified by using ImageJ program. K, The IL‐1β mRNA expression was investigated in HK‐2 cells treated with siRNA for Gadd45β or negative control and stimulated with LPS (1 μg/mL) for the indicated time. **P < .01

To investigate the effect of Gadd45β ablation on inflammatory cytokines in the UUO‐kidney, the expression of pro‐inflammatory cytokines, such as interleukin 1 beta (IL‐1β), interleukin 6 (IL‐6), tumour necrosis factor alpha (TNFα) and inducible nitric oxide synthase (iNOS), was determined by qRT‐PCR. It was found that the levels of all mentioned cytokines significantly increased eight days after UUO in the kidney tissues of both Gadd45β WT and KO mice (Figure 4A‐D). Surprisingly, the mRNA expression levels of pro‐inflammatory markers were significantly lower in the kidneys of Gadd45β KO mice than in those of WT mice, despite the same immune cell infiltration in Gadd45β KO and WT mice (Figure 4A‐D). However, plasma levels of pro‐inflammatory cytokines were not altered by Gadd45β ablation (Figure 4E). We next estimated mRNA expression levels of anti‐inflammatory cytokines such as IL‐4, IL‐5, IL‐10 and IL‐13. There were no significant differences in anti‐inflammatory cytokine levels in the UUO‐kidneys of both Gadd45β WT and KO mice (Figure 4F). Activation markers of T‐lymphocytes including CD25, CD28, CD38 and CD69 were not affected by Gadd45β, suggesting that Gadd45β‐mediated regulation of pro‐inflammatory cytokines mainly occurred in macrophages (Figure 4G). To determine why this discrepancy between immune infiltration and the mRNA expression of genes related to inflammation occurs, BMDMs were isolated and stimulated with lipopolysaccharide (LPS) to induce pro‐inflammatory cytokines. qRT‐PCR analyses indicated that IL‐6 and TNFα gene expression were reduced in response to LPS in BMDMs isolated from Gadd45β KO mice compared with those from WT mice (Figure 4H,I). Consistent with mRNA expression, IL‐1β protein level was reduced in response to LPS in Gadd45β‐deficient BMDMs compared to that in control cells (Figure 4J). Similar IL‐1β gene expression patterns were also observed in HK‐2 cells after LPS treatment (Figure 4K). These data suggest that Gadd45β might be involved in regulating the pro‐inflammatory cytokine expression mediated by UUO.

### 3.5 Effects of Gadd45β deficiency in UUO‐induced renal fibrosis

**Figure 5:**
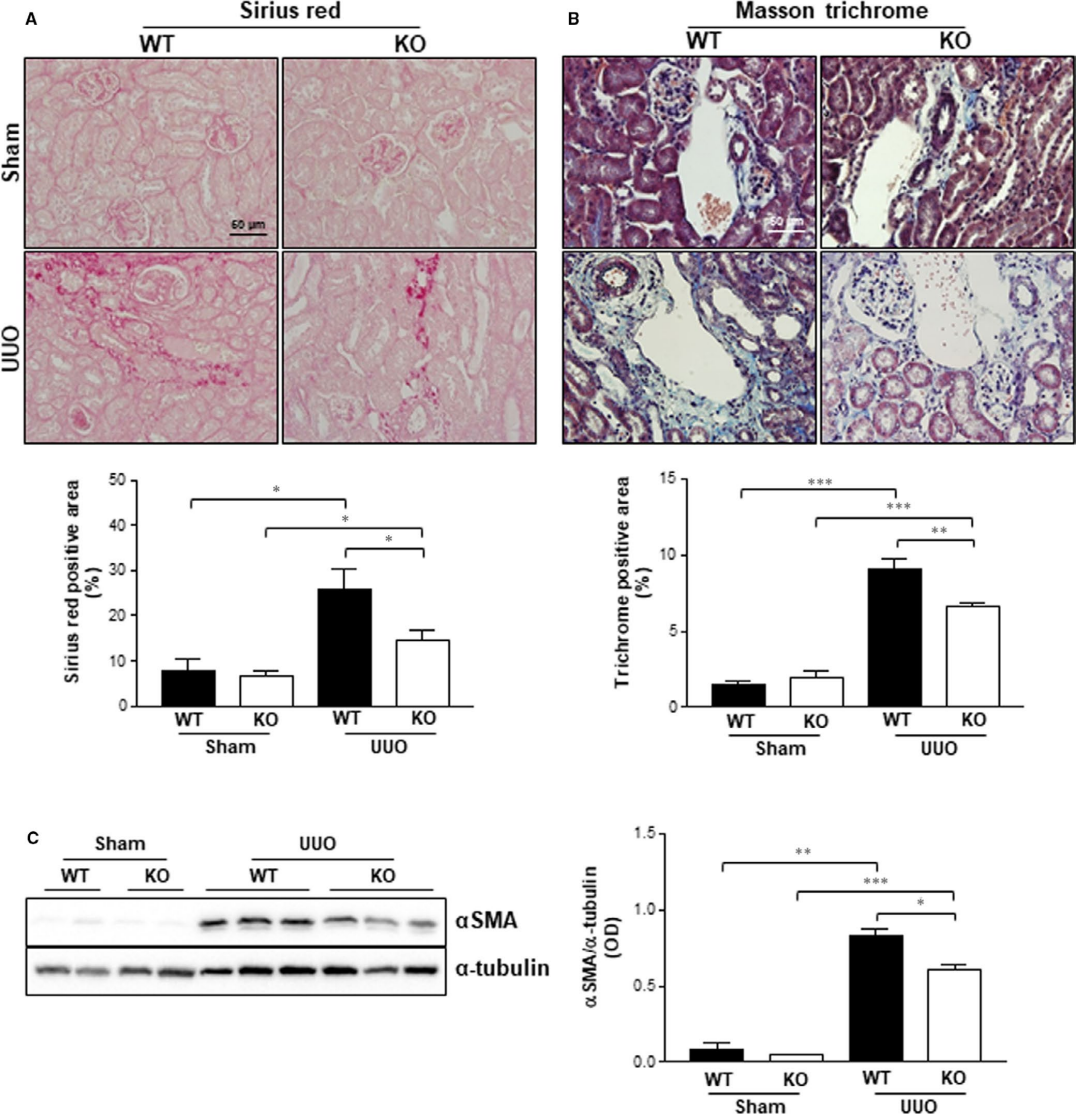
Staining of fibrosis marker and expression of αSMA. Kidney sections from WT‐sham (n = 4), KO‐sham (n = 4), WT‐UUO (n = 5) or KO‐UUO (n = 6) groups were stained with Sirius red (A) and Masson's trichrome stain (B) and positive areas were quantified. The magnification is 40× and the scale bar represents 50 μm. C, The expression of αSMA was analysed by Western blotting, and the optical concentration was normalized by α‐tubulin. *P < .05, **P < .01 or ***P < .001

To investigate the roles of Gadd45β on the fibrotic changes of the kidney after UUO, Sirius red staining was performed and the positive area was quantified to assess the degree of collagen deposition in the kidneys from both genotypes. The sham‐operated kidneys of both genotypes expressed only small areas of collagen deposition (Figure 5A). However, UUO kidneys exhibited a marked increase in Sirius red‐positive areas compared with those of the sham controls (Figure 5A). Interestingly, this UUO‐induced collagen deposition increase was dramatically inhibited in the kidneys of Gadd45β KO mice compared with those of WT UUO (Figure 5A). This result was further confirmed by the evaluation of renal interstitial fibrosis by Masson trichrome staining in mouse kidneys. Similar to the Sirius red staining data, the trichrome positive area significantly increased in the kidneys of both genotypes after UUO, compared with those of the sham‐operated mice (Figure 5B). However, the alteration in UUO was significantly attenuated by the ablation of Gadd45β (Figure 5B). Furthermore, Western blotting demonstrated that UUO‐induced αSMA, the putative myofibroblast marker, was significantly reduced by the ablation of Gadd45β (Figure 5C). These results suggest that Gadd45β is closely involved in the progression of UUO‐induced renal fibrosis.

### 3.6 The roles of Gadd45β on TGF signalling pathways in vitro and in vivo

**Figure 6:**
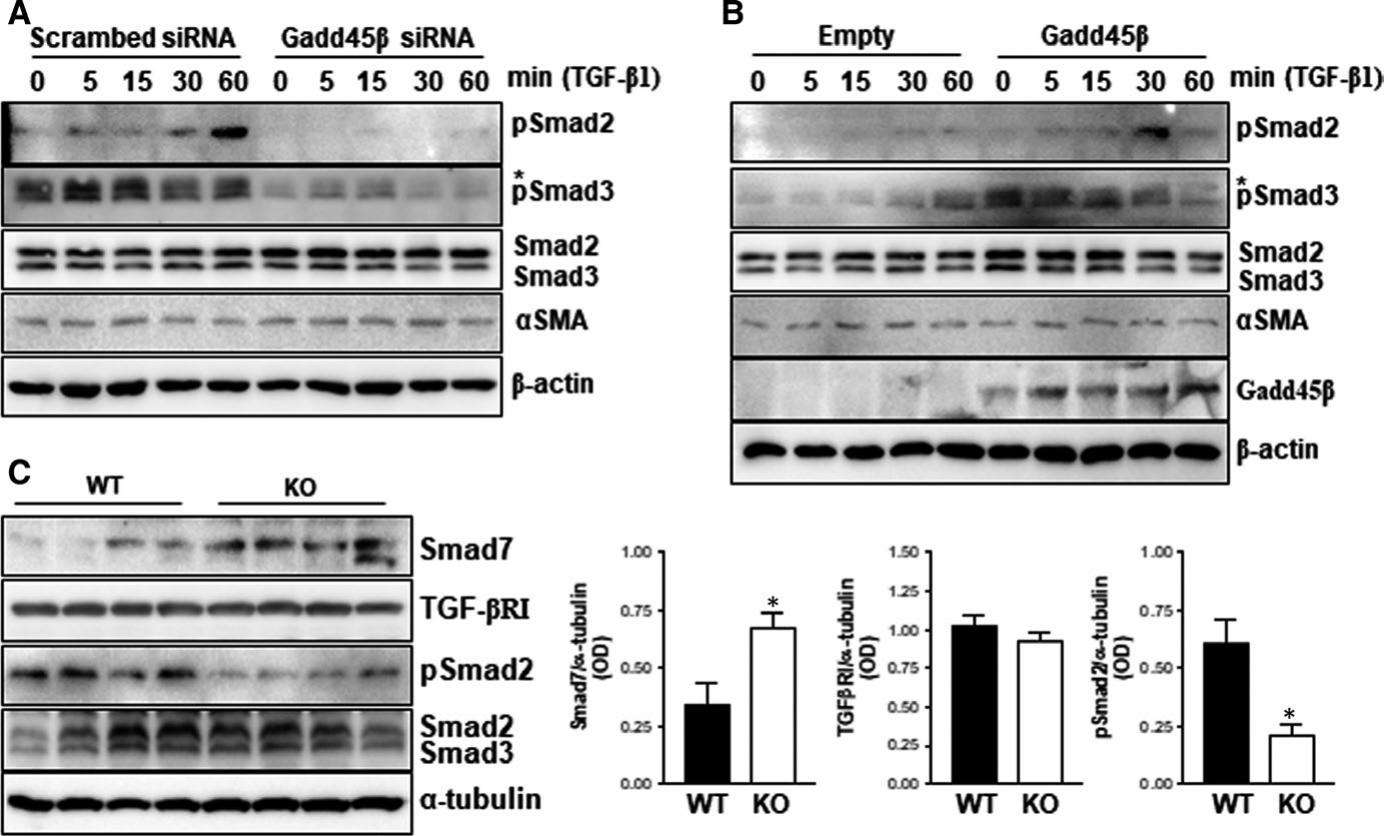
The involvement of Gadd45β in the TGF signalling pathway. HEK293T cells were treated with Gadd45β siRNA (10 nmol/L) and stimulated with recombinant TGF‐β1 (10 ng/mL) in a time‐dependent manner. A, Cell lysates were used for Western blotting using specific antibodies against the indicated molecules. β‐actin was used as a loading control. B, HEK293T cells were transiently transfected with V5 tagged Gadd45β plasmid (500 ng/well) or empty vector. The downstream molecules of TGF signalling were detected by Western blotting. β‐actin was used as a loading control. C, The protein levels and phosphorylation of molecules related to the TGF signalling pathway were determined by Western blot analysis and optical density was expressed by ImageJ program. *P < .05. Data are representative of at least two independent experiments

Previously,^14^ we found that Gadd45β can regulate TGF signalling pathways in intestinal epithelial cells. To determine whether Gadd45β can also regulate TGF signalling in kidneys, HEK 293T cells were incubated with siRNA for Gadd45β and then treated with TGF‐β1 in a time‐dependent manner. The phosphorylation of Smad2 and Smad3, downstream molecules of TGF‐β1, was effectively stimulated by TGF‐β1 treatment. However, TGF‐β1‐induced Smad2/3 activation was inhibited by the silencing of Gadd45β (Figure 6A). In contrast to the silencing data, overexpression of Gadd45β increased the activation of Smad2 after TGF‐β1 stimulation. However, Smad3 phosphorylation was only affected by overexpression of Gadd45β1 and not TGF‐β1 stimulation (Figure 6B). Based on canonical TGF signalling which is initiated by the heterogeneous complexes of Smad2 and Smad3, the phosphorylation of Smad2 in response to TGF‐β1 is critical to Gadd45β‐mediated regulation of TGF signalling pathway in the UUO condition. We further confirmed the roles of Gadd45β on TGF signalling pathways in vivo. Consistent with the in vitro results, phosphorylation of Smad2 was significantly lower in kidney tissues from Gadd45β KO mice than in those from WT mice (Figure 6C). These data suggest that the regulation of TGF‐β signalling by Gadd45β might be a common pathway in various cell types. In our previous study,^14^ we suggested that Gadd45β is involved in TGF signalling pathways via binding to Smad7, resulting in increased protein levels of TGFβRI in intestinal epithelial cell lines. Therefore, we confirmed the levels of TGFβRI and Smad7 in the UUO‐kidneys of both groups. Smad7 protein levels were significantly up‐regulated in the UUO‐kidney of Gadd45β KO mice compared with those in WT mice, whereas TGFβRI protein levels were not different (Figure 6C). These results indicate that Gadd45β can regulate the phosphorylation of Smad2 via modulation of Smad7 stability in kidney after UUO.

## 4 DISCUSSION

The progression of CKD induces renal atrophy, increased leukocyte infiltration and fibrosis in the kidneys.^2^ The UUO model is a standard model for renal fibrosis and chronic kidney failure and mimics a human patient with CKD.^20^ Gadd45β is a small protein with no enzymatic activity of its own.^15^ Instead, its physiological functions are activated by interactions with partner molecules in the nucleus and cytoplasm of cells, and it is involved in cell proliferation, cell death and inflammation.^21^, ^22^, ^23^ Recent studies in experimental animals have shown that Gadd45β is involved in the pathogenesis of various diseases.^14^, ^24^, ^25^ In the present study, Gadd45β KO mice were used and UUO surgery was carried out to induce early stage CKD. A deficiency of Gadd45β led to the lower susceptibility of UUO‐induced nephropathy. The role of Gadd45β on cell proliferation and death, immune cell infiltration and inflammation, and fibrosis was further investigated in mouse kidneys. It was found that cell proliferation increased slightly, but pro‐inflammatory cytokines and renal fibrotic markers were reduced in Gadd45β KO mice after UUO.

Gadd45β is a protein that plays a pivotal role in negative growth control, including growth arrest and apoptotic cell death related to cancer.^23^, ^26^ However, recent studies suggest that its role in growth control is controversial. Indeed, Papa et al^27^ showed that Gadd45β KO mice had significantly reduced proliferation during liver regeneration. In contrast, Tian et al^28^ suggested that the ablation of Gadd45β caused a moderate increase in proliferation following treatment with TCPOBOP. We previously showed that Gadd45β KO mice have delayed wound healing with less proliferation in the intestinal epithelial cells of colon tissues in mice with inflammatory bowel disease.^14^ In the current study, we found higher proliferation in the kidneys of Gadd45β KO mice than those of WT mice after UUO, but no difference was seen in apoptosis. Although we cannot conclude that Gadd45β directly regulates cell proliferation in the kidneys because of compensatory proliferation, our data suggest that the role of Gadd45β on cell proliferation is variable under different cell types and stress conditions.

UUO causes an increase in immune cell infiltration and mRNA expression of pro‐inflammatory cytokines, contributing to the pathogenesis of CKD.^29^, ^30^ Consistent with other studies, the present study showed that UUO significantly increased immune cell in filtration, such as T cells and macrophages, evidenced by a higher percentage of CD3 and F4/80 stained cells. Gadd45β KO mice showed no significant difference in the number of infiltrated immune cells compared with the WT mice. However, the expression of pro‐inflammatory cytokines was lower in the kidneys of Gadd45β KO mice than in those of WT mice. Although we were unable to determine the exact mechanism responsible for the discrepancy between cell infiltration and pro‐inflammatory cytokine levels, BMDMs from Gadd45β KO mice and Gadd45β‐silenced HK‐2 cells showed significantly lower pro‐inflammatory cytokine levels than their controls. These data suggest that the accumulated pro‐inflammatory cytokines in kidney tissues after UUO originate from infiltrated macrophages.

Renal fibrosis is a major pathological sign of CKD caused by various stresses.^31^ TGF‐β1 is a key member of the TGF superfamily.^8^ Various studies have found that the TGF‐β1/Smad signalling pathway contributes to renal fibrosis.^32^, ^33^ Importantly, the suppression of the TGF‐β signalling pathway by pharmacological or genetic means ameliorated the progression of renal fibrosis in patients or experimental animal models.^33^ Recently, we suggested that Gadd45β can bind with Smad7, known as inhibitory Smad, and block the degradation of TGF‐β receptor type 1 mediated by Smurf/Smad7 complex in colon tissue and intestinal epithelial cells.^14^ However, in the present study, we found that Smad7 protein levels in the UUO‐kidney of Gadd45β KO mice were up‐regulated, which subsequently reduce the phosphorylation of Smad2, resulting in less renal fibrosis. We previously showed that TGF‐β1‐induced Smad2 and Smad3 activation was eliminated by silencing Gadd45β, while this effect was reversed by transient up‐regulation in Caco2 cells originating from the intestinal epithelium.^14^ The current study also showed that Gadd45β regulated TGF‐β1‐mediated Smad signalling in kidney cell lines, suggesting that the Gadd45β‐regulated TGF signalling pathway might be involved in the progression of renal fibrosis.

In summary, Gadd45β plays an important role in the progression of kidney disease. Physiologically, Gadd45β is involved in the regulation of cell proliferation, pro‐inflammatory cytokines and renal fibrosis during the progression of CKD. In particular, we found that the TGF signalling pathway is regulated by Gadd45β in kidney fibroblasts. We believe that our novel results contribute to the elucidation of the pathology of CKD, especially in renal fibrosis.

## CONFLICT OF INTEREST

The authors confirm that there are no conflicts of interests.

## AUTHOR CONTRIBUTION

**Sung‐Je Moon:** Data curation (equal); Investigation (lead). **Jae‐Hoon Kim:** Investigation (supporting). **Young‐Keun Choi:** Investigation (supporting). **Chul‐Ho Lee:** Conceptualization (equal); Funding acquisition (equal); Writing‐original draft (equal). **Jung Hwan Hwang:** Conceptualization (equal); Funding acquisition (equal); Investigation (equal); Writing‐original draft (equal).

## Data Availability

The data sets used in this study are available from the corresponding author on reasonable request.
